# mmCSM-NA: accurately predicting effects of single and multiple mutations on protein–nucleic acid binding affinity

**DOI:** 10.1093/nargab/lqab109

**Published:** 2021-11-17

**Authors:** Thanh Binh Nguyen, Yoochan Myung, Alex G C de Sá, Douglas E V Pires, David B Ascher

**Affiliations:** Computational Biology and Clinical Informatics, Baker Heart and Diabetes Institute, Melbourne, Victoria, Australia; School of Chemistry and Molecular Biosciences, The University of Queensland, Brisbane, Australia; Systems and Computational Biology, Bio21 Institute, University of Melbourne, Melbourne, Victoria, Australia; Computational Biology and Clinical Informatics, Baker Heart and Diabetes Institute, Melbourne, Victoria, Australia; Systems and Computational Biology, Bio21 Institute, University of Melbourne, Melbourne, Victoria, Australia; Computational Biology and Clinical Informatics, Baker Heart and Diabetes Institute, Melbourne, Victoria, Australia; School of Chemistry and Molecular Biosciences, The University of Queensland, Brisbane, Australia; Systems and Computational Biology, Bio21 Institute, University of Melbourne, Melbourne, Victoria, Australia; Computational Biology and Clinical Informatics, Baker Heart and Diabetes Institute, Melbourne, Victoria, Australia; Systems and Computational Biology, Bio21 Institute, University of Melbourne, Melbourne, Victoria, Australia; School of Computing and Information Systems, University of Melbourne, Melbourne, Victoria, Australia; Computational Biology and Clinical Informatics, Baker Heart and Diabetes Institute, Melbourne, Victoria, Australia; School of Chemistry and Molecular Biosciences, The University of Queensland, Brisbane, Australia; Systems and Computational Biology, Bio21 Institute, University of Melbourne, Melbourne, Victoria, Australia; Department of Biochemistry, University of Cambridge, Cambridge, UK

## Abstract

While protein–nucleic acid interactions are pivotal for many crucial biological processes, limited experimental data has made the development of computational approaches to characterise these interactions a challenge. Consequently, most approaches to understand the effects of missense mutations on protein-nucleic acid affinity have focused on single-point mutations and have presented a limited performance on independent data sets. To overcome this, we have curated the largest dataset of experimentally measured effects of mutations on nucleic acid binding affinity to date, encompassing 856 single-point mutations and 141 multiple-point mutations across 155 experimentally solved complexes. This was used in combination with an optimized version of our graph-based signatures to develop mmCSM-NA (http://biosig.unimelb.edu.au/mmcsm_na), the first scalable method capable of quantitatively and accurately predicting the effects of multiple-point mutations on nucleic acid binding affinities. mmCSM-NA obtained a Pearson's correlation of up to 0.67 (RMSE of 1.06 Kcal/mol) on single-point mutations under cross-validation, and up to 0.65 on independent non-redundant datasets of multiple-point mutations (RMSE of 1.12 kcal/mol), outperforming similar tools. mmCSM-NA is freely available as an easy-to-use web-server and API. We believe it will be an invaluable tool to shed light on the role of mutations affecting protein–nucleic acid interactions in diseases.

## Graphical Abstract

**Graphical Abstract:**
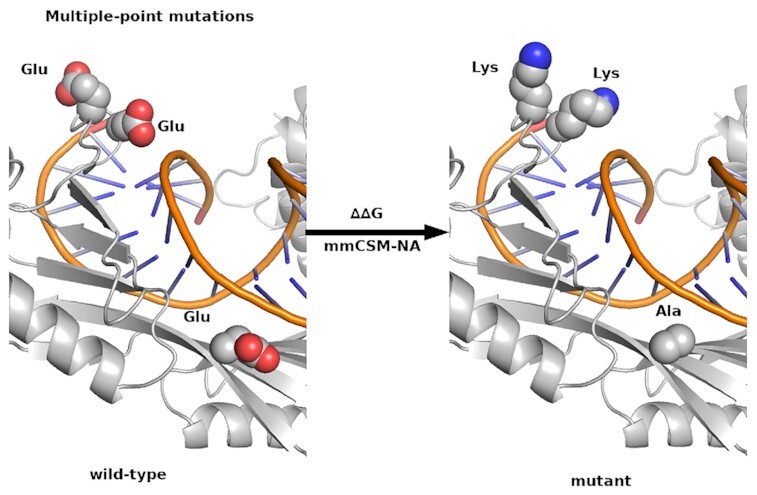
The effects of multiple point mutations on the binding affinity to nucleic acids is accurately predicted by mmCSM-NA.

## INTRODUCTION

The interactions between proteins and nucleic acids play essential roles in crucial biological processes, from gene regulation (1) to replication and transcription (2,3), translation (4), DNA repair (5–8) and DNA packaging (9,10). Missense mutations within nucleic acid binding proteins can lead to a range of diseases, including cancers (11,12), viral infections (13–18) and neurodegenerative disorders (19). With the increasing speed and availability of high-throughput sequencing, there is a pressing need to be able to rapidly evaluate the molecular consequences of novel variants, however, traditional experimental approaches are time-consuming and laborious. Despite increasing interest in the ability to predict the effects of mutations on these protein–nucleic acid (protein–NA) interactions, it remains a significant challenge. In part, this is due to the limited availability of experimental data compared to other protein interactions. Consequently, most developed methods have focused on only the effects of single-point missense mutations and been found to be poorly generalisable (20–22).

We have previously shown that by representing protein structures and their interacting partners as a graph-based signature, it can be used to train predictive models capable of unravelling the link between genotype and phenotype by accurately predicting the effects of mutations on protein folding, stability (23–25), dynamics (26,27) and interactions (28–32). This approach enabled, for the first time, quantitative and scalable assessment of the effects of single-point missense mutations on protein–NA binding affinities (23,33).

**Figure 1.**
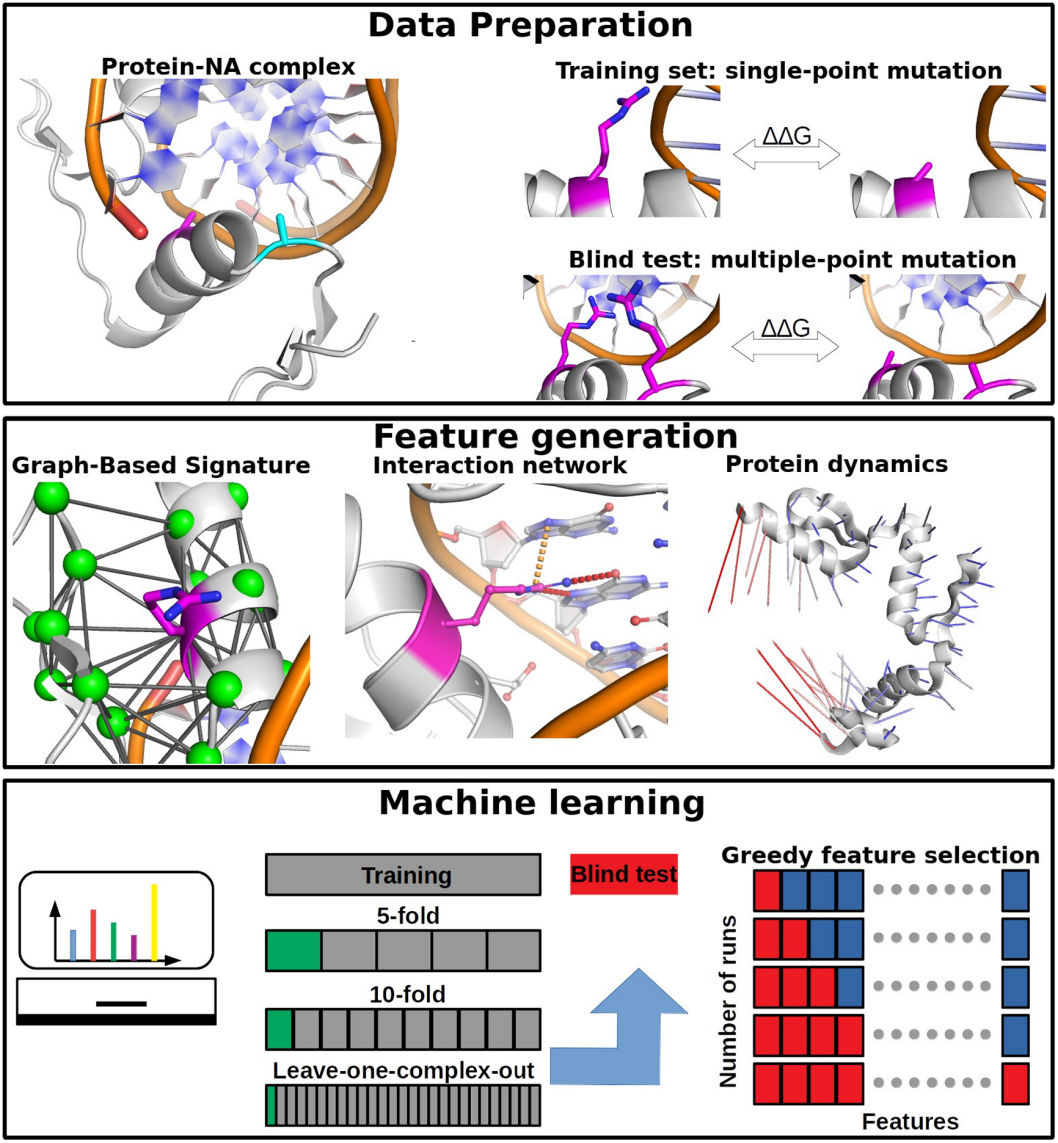
mmCSM–NA workflow and application on real-world data. The method relies on graph-based structural signatures that model distance patterns on the wild-type residue environment. In addition, the effect of mutations was also evaluated using protein dynamics, and interaction network. Complementary information including from mutated residues and predicted protein stability change upon mutation are also used to train and test the predictive models.

Here, we have curated the largest data set with high-quality experimentally characterised effects of mutations on protein–NA affinity to date and developed an optimised version of our graph-based signatures to train and test mmCSM-NA (Figure 1), the first method capable of accurately and scalably predicting the effects of multiple-point mutations on protein–NA binding affinity.

## MATERIALS AND METHODS

### Data sets

Experimentally measured effects of mutations on protein–NA binding affinity were carefully curated, combined and manually corrected for errors from ProNIT 2.0 (34), dbAMERPNI (35) and PrPDH (21). ProNIT provides the effects of mutations as experimental thermodynamic values given in the form of the difference in Gibbs free energy of binding, ΔΔ*G* (in kcal/mol), or kinetic equilibrium constant, *K*_D_ (in molar). dbAMERPNI comprises of >570 alanine scanning data points with experimental thermodynamic values. PrPDH is a data set consisting of 214 mutations of protein-DNA interactions with experimental ΔΔ*G* values. In total, our combined data set has 856 single-point mutations across 151 unique protein–NA complexes (484 neutral, 349 decreasing and 23 increasing affinities) and 141 multiple-point mutations on 25 protein–NA complexes (77 decreasing, 62 neutral and 2 increasing affinities). The target outcome for mmCSM-NA is the changes in Gibbs binding free energy (or ΔΔ*G*). ΔΔG was defined as the difference between wild-type and mutant (ΔΔ*G* = Δ*G*_wt_ – Δ*G*_mut_), where negative values of ΔΔ*G* correspond to mutations decreasing protein–NA affinity and positive values to those increasing affinity.

Only single-point missense mutations were considered within our training data set, which was further divided into four distinct subclasses based on the interacting partners: double-stranded DNA (dsDNA; 461 single-point mutations across 81 complexes), single-stranded DNA (ssDNA; 60 single-point mutations across 13 complexes), double-stranded RNA (dsRNA; 188 single-point mutations across 30 complexes), and single-stranded RNA (ssRNA; 147 single-point mutations across 27 complexes). Structures where separate single-stranded nucleic acids formed a pair were considered as double-stranded.

The Gibbs free energy can be presented as a thermodynamic state function, *i.e*. the Gibbs free energy only depends on the beginning and end states. In other words, the change in Gibbs free energy of a mutation from the wild-type to mutant (ΔΔ*G*_wt→mut_) is equivalent in intensity but with opposite sign of the reverse mutation, *i.e*. from the mutant to wild-type (ΔΔ*G*_mut→wt_ = –ΔΔ*G*_wt→mut_). The use of hypothetical reverse mutations has been proposed to help balance naturally imbalanced thermodynamic data sets (36). One limitation of this approach, however, is that mutations leading to large changes in ΔΔ*G* are more likely to lead to larger changes in protein structure, which are unlikely to be accurately modelled. To avoid this, we generated mutant structures and have only considered reverse mutations where their ΔΔ*G*_wt→mut_ ≥ −2 kcal/mol. In total, we considered reverse mutations of 699 single-point mutations of 136 protein–NA complexes, including dsDNA (391 single-point mutations across 72 complexes), ssDNA (45 single-point mutations across 11 complexes), dsRNA (158 single-point mutations across 29 complexes) and ssRNA (105 single-point mutations across 24 complexes). The addition of 699 reverse mutations into 856 experimental single mutations results in a fairly balanced training set of 1555 single-point mutations (Supplementary Figure S1).

All 141 multiple-point mutations across 25 protein–NA complexes were used as an independent blind test. They included dsDNA (113 multiple-point mutations across 18 complexes), ssDNA (12 single-point mutations across 3 complexes), dsRNA (12 single-point mutations across 3 complexes) and ssRNA (4 single-point mutations across 1 complex). Data sets used are available at http://biosig.unimelb.edu.au/mmcsm_na/datasets.

### Mutation modelling and feature engineering

#### Modelling mutant structures

Mutant structures for single-point and multiple-point mutations were built using Modeller (v.9.25) (37). The conformation of the mutant side chain was then optimized by conjugate gradient and refined by molecular dynamics simulations using default parameters from *mutate_model.py* in the Modeller website.

#### Feature engineering

Five classes of features were extracted from protein-NA complexes (Supplementary Table S1).

- *Graph-based signatures*. The mCSM graph-based signatures have been applied to represent both the environment of the wild-type residue of the protein and the nucleic acid by calculating the physicochemical and geometry of their interactions (23). The signature defines atoms as nodes and their interactions as edges. Nodes are labelled based on the seven physicochemical properties (aromatic, hydrophobic, negative, positive, hydrogen bond donor, hydrogen bond acceptor, and neutral) of the amino acid residues/nucleic acids (*i.e*. pharmacophores). Nucleic acid nodes were labeled based on nucleic acid pharmacophores considering the base ring (purines or pyrimidines), and nucleic acid components (phosphate, sugar and base) and protein nodes as performed previously (33). By using the cutoff scanning matrix algorithm, residue environments are represented as cumulative distributions of distances.
- *Physicochemical properties of protein amino acid residues*. The statistical quasi-chemical potential with the composition-corrected pair scale and two substitution matrices from AAindex database (38) were used to calculate the effect of mutations.
- *Normal mode analysis (NMA)*. The dynamics aspects of the mutation effects were characterised by the vibrational entropy change of the protein–NA **compelx** using DynaMut (26).
- *Protein-nucleic acid interaction network*. The non-covalent interactions between protein and nucleic acid in both wild-type and mutated complexes were calculated using Arpeggio(39). Thirteen interaction types are considered: clash, covalent, Van der Waals, clash in Van der Waals, hydrogen bond, weak hydrogen bond, proximal, halogen bond, aromatic, ionic, carbonyl, hydrophobic and metal interactions.
- *Nucleic acid type*. Four types of nucleic acids, ssRNA, dsRNA, ssDNA and dsDNA were considered in our prediction model. The type was split into RNA/DNA and double/single-stranded as binary variables.

Multiple mutation features were calculated as either the average or accumulation of the respective single point mutations.

### Machine learning approach

A range of supervised machine learning algorithms for regression available on Scikit-Learn version 0.20.3 (40), were evaluated under a group-based 5-, 10- and 20-fold cross-validation procedure and evaluated against the blind test set. To reduce issues with redundancy, the forward mutations and their respective reverse counterparts were kept in the same set.

Under this evaluation framework, the following learning algorithms were analyzed: Gradient Boosting, Extreme Gradient Boosting, Random Forest, Extremely Randomized Trees, AdaBoost, K-Nearest Neighbour, Support Vector Regressor, Gaussian Processes and Neural Networks (with a standard Multi-Layer Perceptron). Models were optimized using a traditional bottom-up greedy feature selection approach. The best performing model, based on Pearson's correlation coefficient (PCC) and root mean square error (RMSE) on the training set, was extremely randomized trees. Other performance metrics were also considered and reported including Spearman's and Kendall's correlations; accuracy, F1 and Matthew's correlation coefficient (MCC) for classification-by-regression analyses. 13 features were selected as representatives for the predicted model.

### Web-server

The server front end was built using the Bootstrap framework version 3.3.7, whereas the back-end was built using Python via the Flask framework (Version 0.10.1), on a Linux server running Apache.

## RESULTS

### Data set analysis

The distributions of the changes in Gibbs free energy, ΔΔ*G*, in the 856 single-point mutations and 141 multiple-point mutations having experimental values show that majority of the mutations are destabilizing (Supplementary Figure S1). To avoid introducing potential bias into the final model, hypothetical reverse mutations of the single-point missense mutations were included in the training set. We next analyzed the distribution of different amino acid types on wild-type and on mutated residues. The majority of the 856 experimentally-determined single-point mutations involve positively charged residues, namely Arg and Lys (17%, and 14%, respectively) (Supplementary Figure S2A), followed by negatively charged, and aromatic residues, namely Tyr, Asp, Phe and Glu (8%, 7%, 6% and 6%, respectively). Most of the residues involved in single-point mutations are mutated to Ala (74%), as a reflection of alanine scanning efforts, while other amino acid types account for no more than 2%. Similarly, the majority of 141 multiple-point mutations consist of 16% of Arg and 19% of Lys (Supplementary Figure S2B), followed by Ser (11%), His (7%), Asp (6%) and Glu (6%). Most of the residues in multiple-point mutations are also mutated to Ala (41%), followed by Asn (7%), Lys (6%), Glu (6%), Arg (5%) and Gly (5%), while other amino acid types account for <5%.

### Performance on single-point mutations

**Figure 2.**
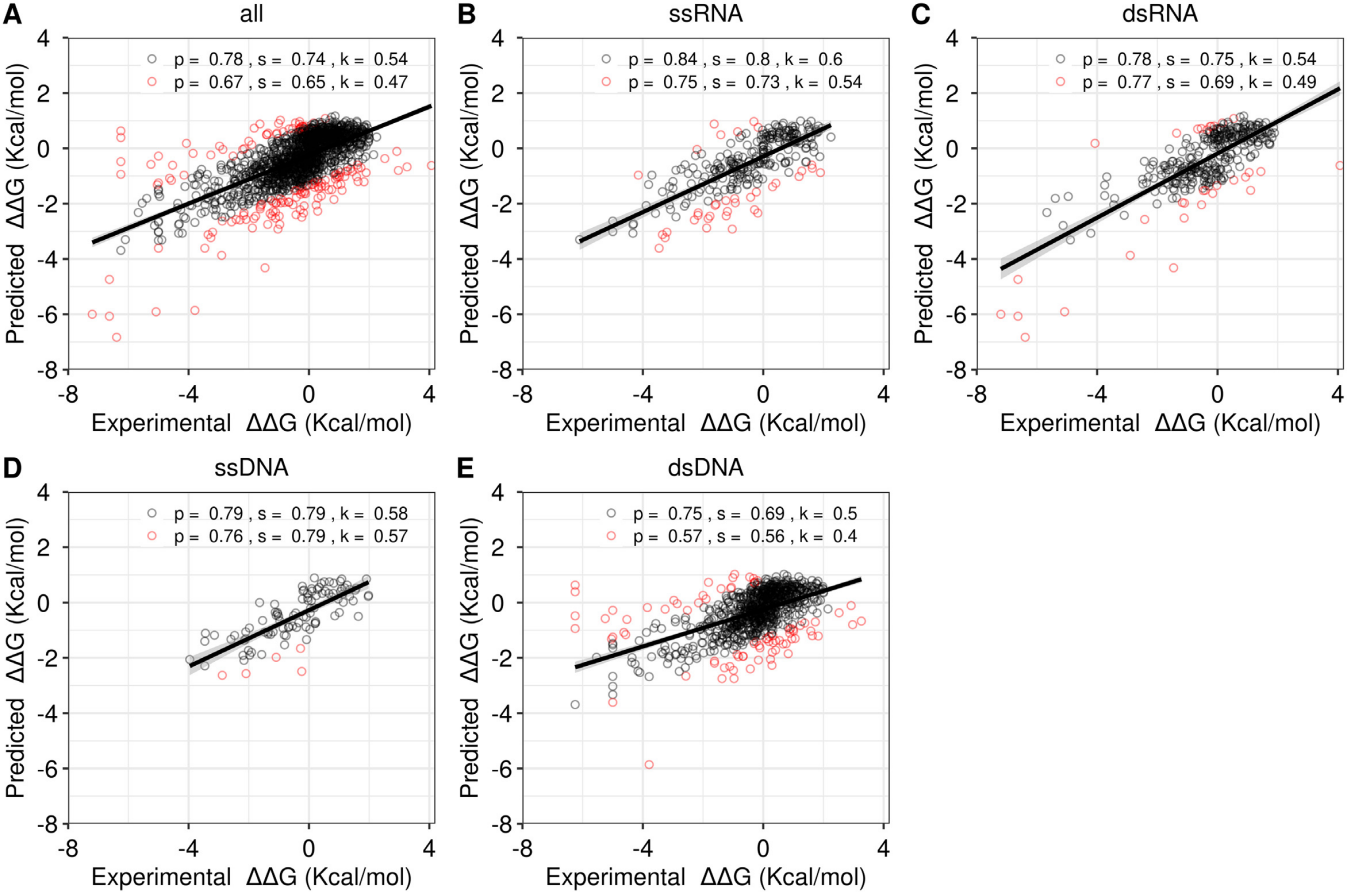
Regression plot between the experimental and predicted changes in binding affinity (in kcal/mol) during cross-validation. mmCSM–NA obtained a Pearson's correlation of 0.67 across the original dataset (**A**). The performance of the model against complexes containing ssRNA (**B**), dsRNA (**C**), ssDNA (**D**) and dsDNA (**E**) are shown, highlighting the accuracy and applicability of mmCSM–NA to handle all different types of protein–NA complexes. The overall Pearson correlation coefficients, including outliers, is shown in red; with the correlation after removing outliers shown in black. The Pearson's, Spearman’ and Kendall's correlations are written in abbreviation as p, s and k, respectively

The performance of mmCSM-NA was evaluated via multiple forms of cross-validation. Using 5-, 10- and 20-fold cross-validation, for single-point mutations mmCSM-NA achieved Pearson's, Spearman's, and Kendall's correlations of up to 0.67, 0.65 and 0.47 respectively, with small deviations across repetitions (<0.01; Figure 2A) and RMSE of 1.06 kcal/mol. Performance increases significantly on 90% of the data, removing 10% of worst predicted data points, achieving Pearson's, Spearman's, and Kendall's correlations of up to 0.78, 0.74, and 0.54 (RMSE = 0.84 kcal/mol). Looking closer at the outliers revealed most of them were associated with extreme values (ΔΔ*G* either lower than −3 kcal/mol or higher than 3 kcal/mol). Reassuringly, however, their direction of change was still predicted correctly.

In addition to the overall model performance, we next considered the performance of our final model on each nucleic acid type. Pearson's correlation values of mmCSM-NA on the protein-NA complexes are 0.75, 0.77, 0.76, and 0.57, for ssRNA, dsRNA, ssDNA and dsDNA, respectively (Figure 2B–E). After 10% outlier removal, the corresponding Pearson's correlations increased to 0.84, 0.78, 0.79 and 0.75.

We further assessed the robustness of the mmCSM-NA model on a low-redundancy setting, via leave-one-complex-out cross-validation. mmCSM-NA was able to achieve Pearson's, Spearman's, and Kendall's correlations of up to 0.50, 0.57 and 0.40, respectively (RMSE = 1.24 kcal/mol), still consistent with its original performance.

As many experimental screening efforts have focussed on alanine scanning, the performance of the mmCSM-NA model on different types of mutations was evaluated. While the Pearson's correlation for mutations to alanine was 0.55 (RMSE = 1.09 kcal/mol), interestingly, mutations to other residues had a Pearson's correlation of 0.61 (RMSE = 1.26 kcal/mol) (Supplementary Table S2), indicating that our approach has a comparable performance on non-alanine scanning mutations.

We also analysed the dependency of the performance of the mmCSM–NA model on the distance between NA and protein atoms. We used a cutoff of 8 Å to distinguish proximal from distal mutations (Supplementary Figure S3). mmCSM–NA was able to achieve comparable performance between mutations within and beyond 8 Å from the interface, achieving Pearson's correlations up to 0.58 (RMSE = 0.90 kcal/mol) and 0.69 (RMSE = 1.13 kcal/mol), respectively (Supplementary Table S3).

To further assess the performance of the developed method, we evaluated its ability to correctly identify the direction of affinity change (*i.e*. either increase or decrease affinity). For this purpose, we removed all the single-point mutations that were considered as neutral (−0.5 ≤ ΔΔ*G* ≤ 0.5). The mmCSM–NA model was able to accurately classify the direction of affinity change due to mutations, with accuracy, F1 and MCC of up to 0.86, 0.88 and 0.70, respectively, achieving an AUC of 0.91 (Supplementary Figure S4).

### Benchmarking with other available methods

Most efforts to build computational models exploring the effects of mutations on protein–NA binding affinity have focused on predicting residues significantly contributing to binding affinity (*i.e*. hot-spot residues). We, therefore, sought to compare the ability of mmCSM–NA to identify these hot-spots with other available tools. For this task, hot-spots were considered as residues where mutation to Alanine results in complete disruption of the interaction.

Three methods were used in a comparison with mmCSM-NA, namely, iPNHOT (20), PrabHot (22) and PrPDH (21), which predict the hot-spots of protein–NA, protein–RNA and protein–DNA complexes, respectively. Unlike mmCSM-NA, these methods are limited to identifying hot-spot residues in close proximity to the binding site and are not capable of predicting quantitative effects.

Different approaches have used different cutoff values to define hot-spots/non-hot-spots. For direct comparison purposes, we therefore used the same threshold defined in each of the respective methods. For example, in the case of iPNHOT, we used the cutoff of −2 kcal/mol, while in the cases of PrPDH and PrabHot, we used the cutoff of −1 kcal/mol.

We assessed the ability of the methods to identify hot-spots by using all mutations to Alanine that had available experimental values in our curated data set as a blind test. mmCSM-NA outperformed all three methods, achieving MCCs of 0.37, 0.39 and 0.28 when using different cutoffs to define hot-spots in comparison with iPNHOT (MCC = 0.28), PrPDH (MCC = 0.31) and PrabHot (MCC = 0.07), respectively, as shown in Table 1.

**Table 1.** Benchmark with other servers that predict the hot-spots and non-hot-spots in protein–NA complexes^a^

	Cutoff (kcal/mol)	Method	SEN	SPE	PRE	ACC	F1-Score	MCC	*P*-value
DNA+RNA	−2	mCSM-NA	0.31	0.96	0.64	0.85	0.42	0.37	<0.001
		iPNHOT	0.37	0.90	0.43	0.80	0.40	0.28	
DNA	−1	mCSM-NA	0.63	0.77	0.61	0.72	0.62	0.39	0.006
		PrPDH	0.48	0.81	0.59	0.70	0.53	0.31	
RNA	−1	mCSM-NA	0.54	0.73	0.63	0.64	0.58	0.28	<0.001
		PrabHot	0.69	0.37	0.48	0.52	0.57	0.07	

^a^Non-predicted residues in other servers are considered as non-hot-spots.

### Performance on multiple-point missense mutations

The predicted model was evaluated using a blind data set of multiple-point mutations. Although the training set did not contain any multiple-point mutations, mmCSM-NA was able to accurately predict the change in Gibbs binding free energy of multiple-point mutations with Pearson's, Spearman's and Kendall's correlations of 0.65, 0.56 and 0.41, respectively (RMSE = 1.12 kcal/mol) (Supplementary Figure S5). This performance shows the ability of the method to predict multiple-point mutations using the information from only single-point mutations. To further assess the performance of the developed method, we evaluated its ability to correctly identify the direction of affinity change (*i.e*. either increase or decrease affinity). For this purpose, we removed all the multiple-point mutations that were considered as neutral (−0.5 ≤ ΔΔ*G* ≤ 0.5). The mmCSM-NA model was able to accurately classify the direction of affinity change due to mutations, with accuracy, F1, and MCC of up to 0.96, 0.98 and 0.55, respectively and AUC of 0.90.

The combination of multiple point mutations can be either additive, where the overall effect equals the sum of contributions of individual mutations, or synergistic, where there are compensatory effects between the mutations. We therefore assessed the performance of mmCSM–NA on additive and synergistic multiple-point mutations. Across our dataset of multiple point mutations, we had 104 mutations across 17 complexes that had experimental information available on all the mutations in isolation, in addition to the multiple-point mutation construct. Multiple-point mutation constructs were defined as additive mutations when the difference between the overall effect and the contributions of the individual mutations effects was either lower than 0.2 kcal/mol (leading to 39 additive mutations) or varied by <10% (leading to 12 additive mutations). Mutations were considered synergistic otherwise, leading to 65 and 92 synergistic mutations, respectively. Our method performed consistently on both synergistic and additive mutations, achieving Pearson's correlations of 0.60 and 0.63 (for additive and synergistic, respectively, for the ±10% criteria) and 0.61 and 0.59 (for additive and synergistic, respectively, for the ±0.2 kcal/mol criteria) (Supplementary Figure S6).

### Assessing feature importance

The final mmCSM–NA model is composed of a diverse set of 13 features. We assessed their relative contribution to the model (Supplementary Figure S7) using SHAP (41). This highlighted that the 3D environment of the mutation (graph-based signatures in addition to changes in vibration entropy and protein dynamics) and its interactions with the nucleic acid (Arpeggio interactions and distance to nucleic acid) were the most important contributors to overall performance. In addition, amino acid similarity matrices from AAIndex and the nature of the nucleic acid (DNA or RNA) were also important considerations.

### Web-server

We have made mmCSM-NA available to the research community as an easy-to-use web-server. To perform a prediction, users need to provide either a PDB file or a PDB code of the protein–NA complex and specify the type of nucleic acid (ssRNA, dsRNA, ssDNA and dsDNA) (Supplementary Figure S8). Users can select either ‘Prediction Mode’, where users supply a list of mutations, or ‘Design Mode’, where the server performs saturation mutagenesis across all residues within 8Å of the protein–nucleic acid interface. With Prediction Mode, users can either type or upload a list of mutations. The point mutation should contain the chain identifier of the wild-type residue in the protein, its single letter code, its corresponding residue number, and the single letter code of the mutant residue. The chain identifier and the single letter code should be separated by a space. For multiple mutations, these can be listed sequentially using ‘;’ as a separator, while distinct constructs should be separated by different lines. The ‘Prediction Mode’ is also available via RESTful Application Programming Interfaces (APIs) and examples of its usage are described in the web-server.

mmCSM–NA predicts the numerical values of the change in Gibbs binding free energy (ΔΔ*G* in kcal/mol) (Supplementary Figure S9) in tabular format, which is made available to download as a comma-separated file. A negative value corresponds to the destabilizing effect, while a positive value presents the stabilizing effect.

The users can visualize their uploaded PDB file with its wild-type residue environment from the server using GLmol molecular viewer (Supplementary Figure S9), and a Pymol session file showing all the intra- and inter-molecular interactions made by the wild-type residue, calculated by Arpeggio, is available for download and viewing in Pymol for preparation of publication quality figures and to allow further analysis.

## DISCUSSION

In this work, we present mmCSM-NA as a new method that can, for the first time, rapidly and accurately predict the effects of single- and multiple-point mutations on the protein-nucleic acid binding affinity. Our approach, which was developed using graph-based signatures, was robust across multiple forms of validation; and despite only being trained using single-point mutations was able to accurately predict the effects of multiple-point mutations on protein–nucleic acid binding affinity.

We benchmarked our methods with other available methods, particularly iPNHOT, PrPDH and PrabHot. The benchmark showed that mmCSM–NA outperformed those methods, with the advantage of predicting quantitatively the change in binding free energy even for remote regions of the binding site.

We believe mmCSM–NA will be of great value for the study and design of mutations affecting protein–nucleic acid interactions.

## DATA AVAILABILITY

A user-friendly web-server implementing the mmCSM–NA’s predictive model and all curated data is freely available at: http://biosig.unimelb.edu.au/mmcsm_na/.

## Supplementary Material

lqab109_Supplemental_File
