# Supplementary material for: mmCSM-NA: accurately predicting effects of single and multiple mutations on protein–nucleic acid binding affinity
Source: NAR Genom Bioinform. 2021 Nov 17;3(4):lqab109. doi: 10.1093/nargab/lqab109 (PMC8600011; doi:10.1093/nargab/lqab109)
Supplement: lqab109_Supplemental_File [file lqab109_supplemental_file.doc]

**SUPPLEMENTARY MATERIALS**

**mmCSM-NA: accurately predicting effects of single and multiple mutations
on protein‒nucleic acid binding affinity**

Thanh Binh Nguyen^1,2,3^, Yoochan Myung^1,3^, Alex G. C. de Sá^1,2,3^, Douglas E. V. Pires^1,3,4,*^, David B. Ascher^1,2,3,5,*^

^1^ Computational Biology and Clinical Informatics, Baker Heart and Diabetes Institute, Melbourne, Victoria

^2^ School of Chemistry and Molecular Biosciences, The University of Queensland, Brisbane, Australia

^3^ Systems and Computational Biology, Bio21 Institute, University of Melbourne, Melbourne, Victoria

^4^ School of Computing and Information Systems, University of Melbourne, Melbourne, Victoria

^5^ Department of Biochemistry, University of Cambridge, Cambridge, UK

*To whom correspondence should be addressed D.B.A. Tel: +61 90354794; Email: [david.ascher@unimelb.edu.au](mailto:david.ascher@unimelb.edu.au). Correspondence may also be addressed to D.E.V.P. [douglas.pires@unimelb.edu.au](mailto:douglas.pires@unimelb.edu.au).

**SUPPLEMENTARY TABLES**

**Table S1.** Features and methods that used to generate them for the training and blind test data set.

| **Feature** | **Method used** |
| --- | --- |
| Graph-based signatures (protein) | mCSM-Stability |
| Graph-based signatures (nucleic acids) | mCSM-NA |
| Protein dynamics | Dynamut |
| Non-covalent interactions | Arpeggio |
| Residue depth | BioPython |
| Solvent Accessible Area | BioPython |
| Amino acid properties | Aaindex |
| Conservation | PAM30, BLOSUM62 |

**Table S2. Performance of mmCSM-NA stratified by wild-type and mutant amino acids residue types, considering forward and reverse mutations.**

| 10-fold cross validation results using the complete set of mutations (1555 mutations) | | |
| --- | --- | --- |
| Class (Wild-type to Mutant) | Pearson's correlation,  RMSE (Kcal/mol),  (Number of mutations) | |
|  | Forward & reverse mutations | Forward mutation |
| any to any | 0.67  RMSE = 1.06  (1555) | 0.57  RMSE = 1.19  (856) |
| non-Ala to Ala (nonA2A) | 0.55  RMSE = 1.09  (639) | 0.55  RMSE = 1.08  (633) |
| Ala to non-Ala (A2nonA) | 0.26  RMSE = 0.84  (532) | 0.64  RMSE = 2.51  (8) |
| non-Ala to non-Ala (nonA2nonA) | 0.61  RMSE = 1.26  (384) | 0.64  RMSE = 1.40  (215) |

**Table S3. Performance of mmCSM-NA stratified by the distance between NA and proteins atomss, considering forward and reverse mutations.**

| 10-fold cross validation results using the complete set of mutations (1555 mutations) | | |
| --- | --- | --- |
| NA-protein distance | Pearson's correlation,  RMSE (Kcal/mol),  (Number of mutations) | |
|  | Forward & reverse mutations | Forward mutation |
| any | 0.67  RMSE = 1.06  (1555) | 0.57  RMSE = 1.19  (856) |
| > 8 Å | 0.58  RMSE = 0.90  (543) | 0.49  RMSE = 0.99  (287) |
| ≤ 8 Å | 0.69  RMSE = 1.13  (1012) | 0.57  RMSE = 1.28  (569) |

**Table S4.** Pearson’s correlations before (redundant) and after (non-redundant) removal of the single mutations (in training set) that present in multiple mutations (blind test set).

|  | Training | Training (90%) | Blind test | Blind test (90%) |
| --- | --- | --- | --- | --- |
| Redundant | 0.67 | 0.78 | 0.65 | 0.74 |
| Non-redundant | 0.64 | 0.75 | 0.53 | 0.69 |

**SUPPLEMENTARY FIGURES**


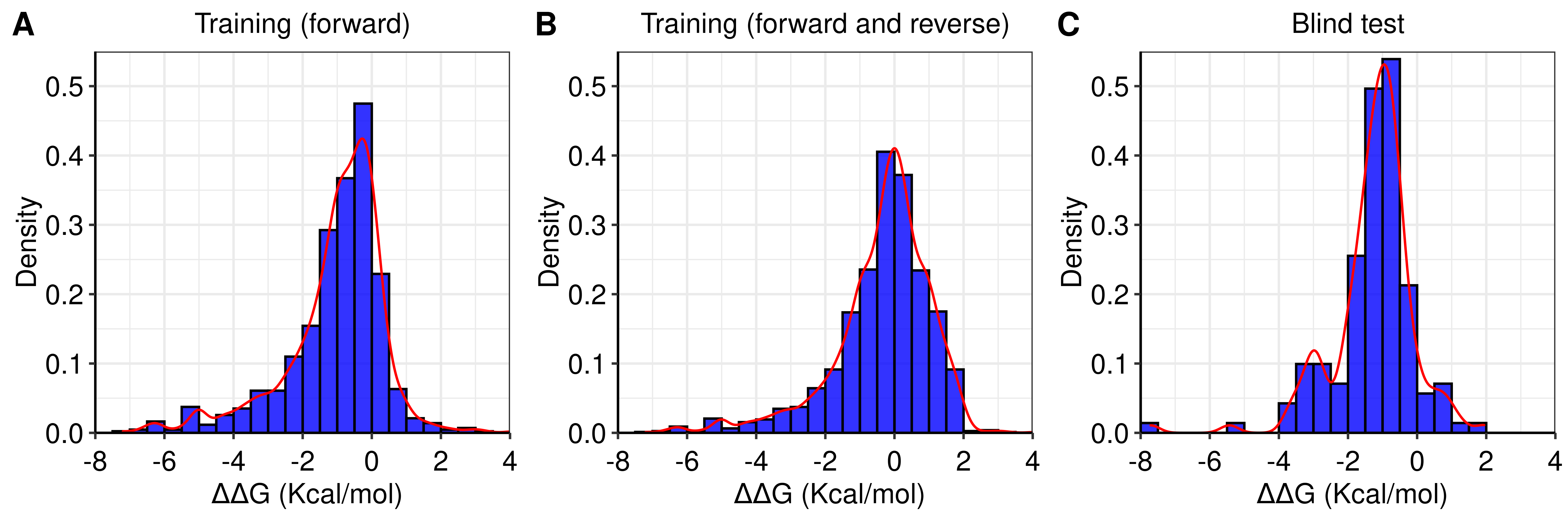


**Figure S1.** Histogram of experimental binding free energies of the forward single-point mutations in training (A), all single-point mutations in training (B), and the blind test (C).


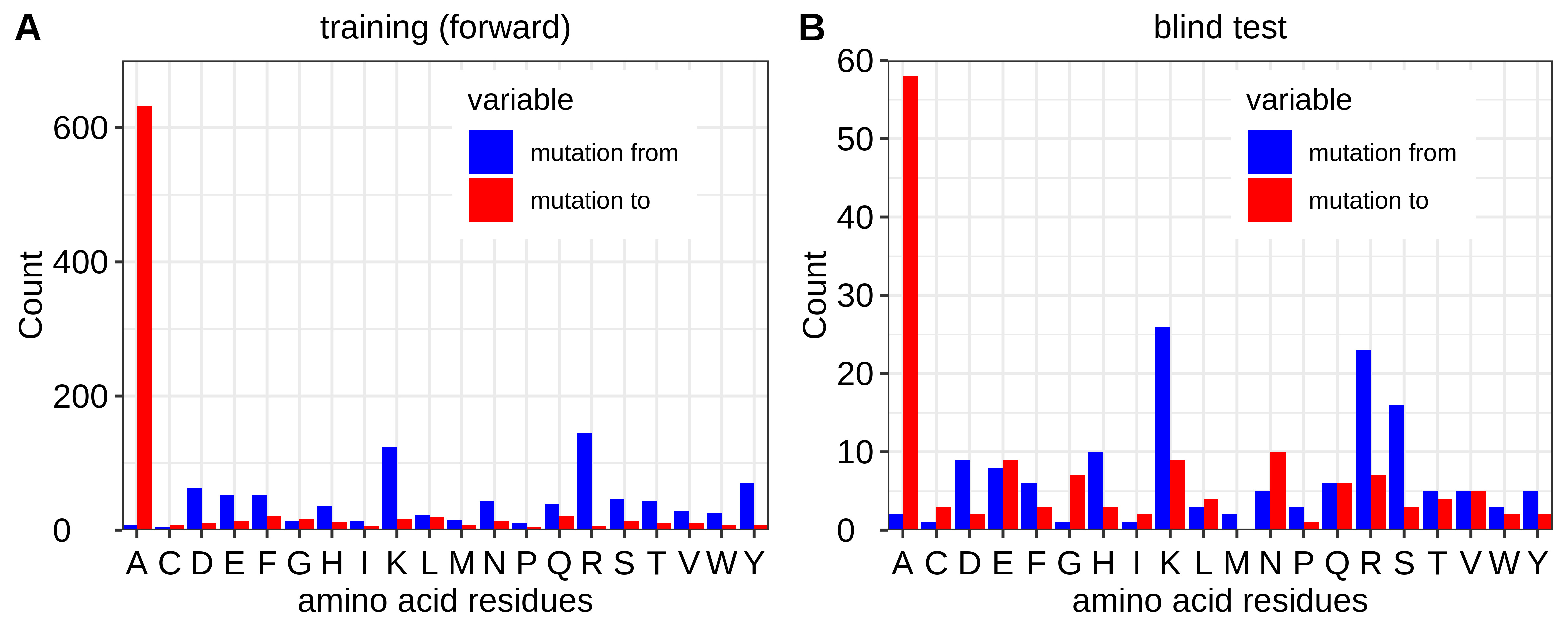
**Figure S2.** Frequency of amino acid types in the combined data set of 856 single-point mutations (A) and 141 multiple-point mutations (B). Blue colour represents the amino acid type of the wild-type structure, while red represents the amino acid type of the mutants.


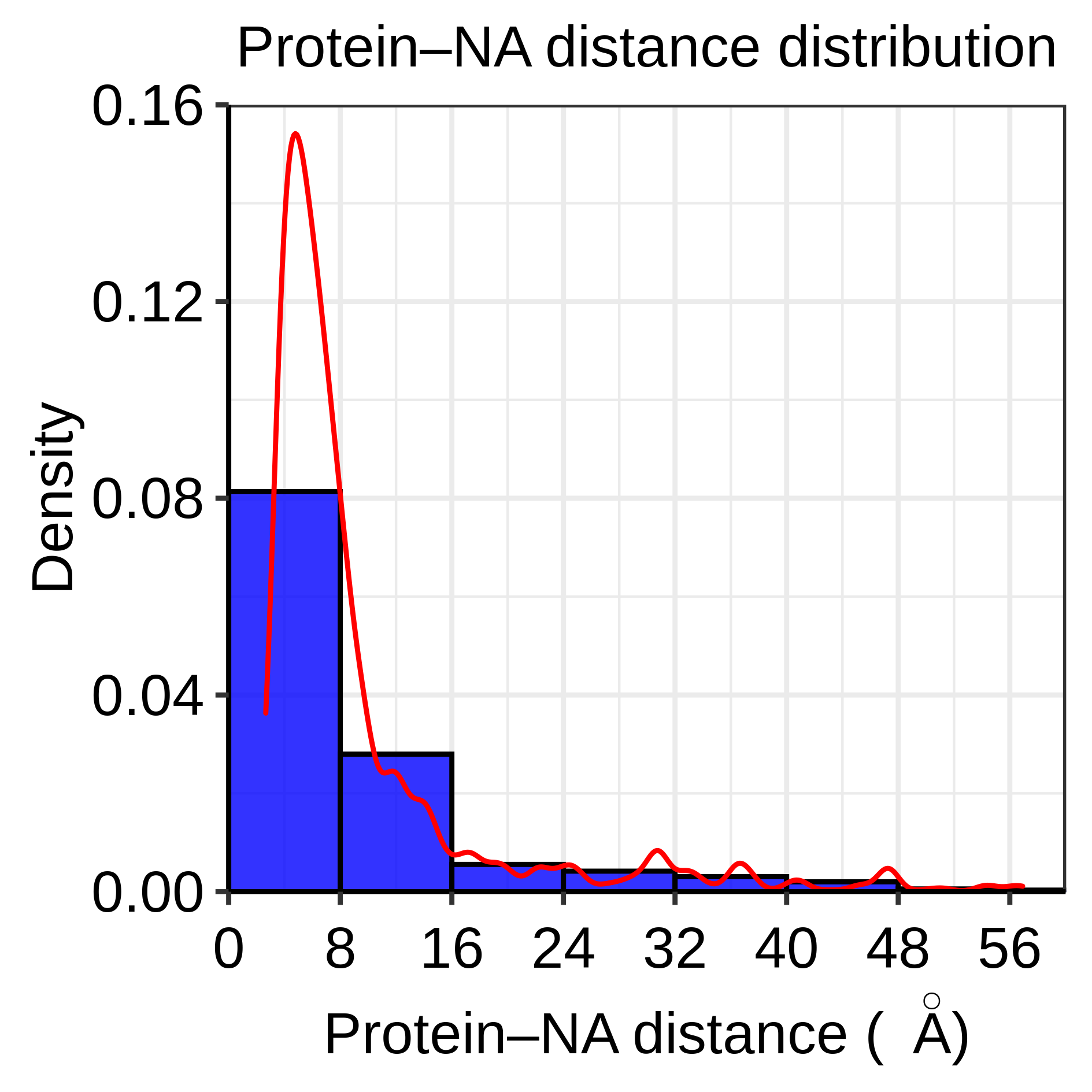
**Figure S3.** Distribution of protein‒NA distance.


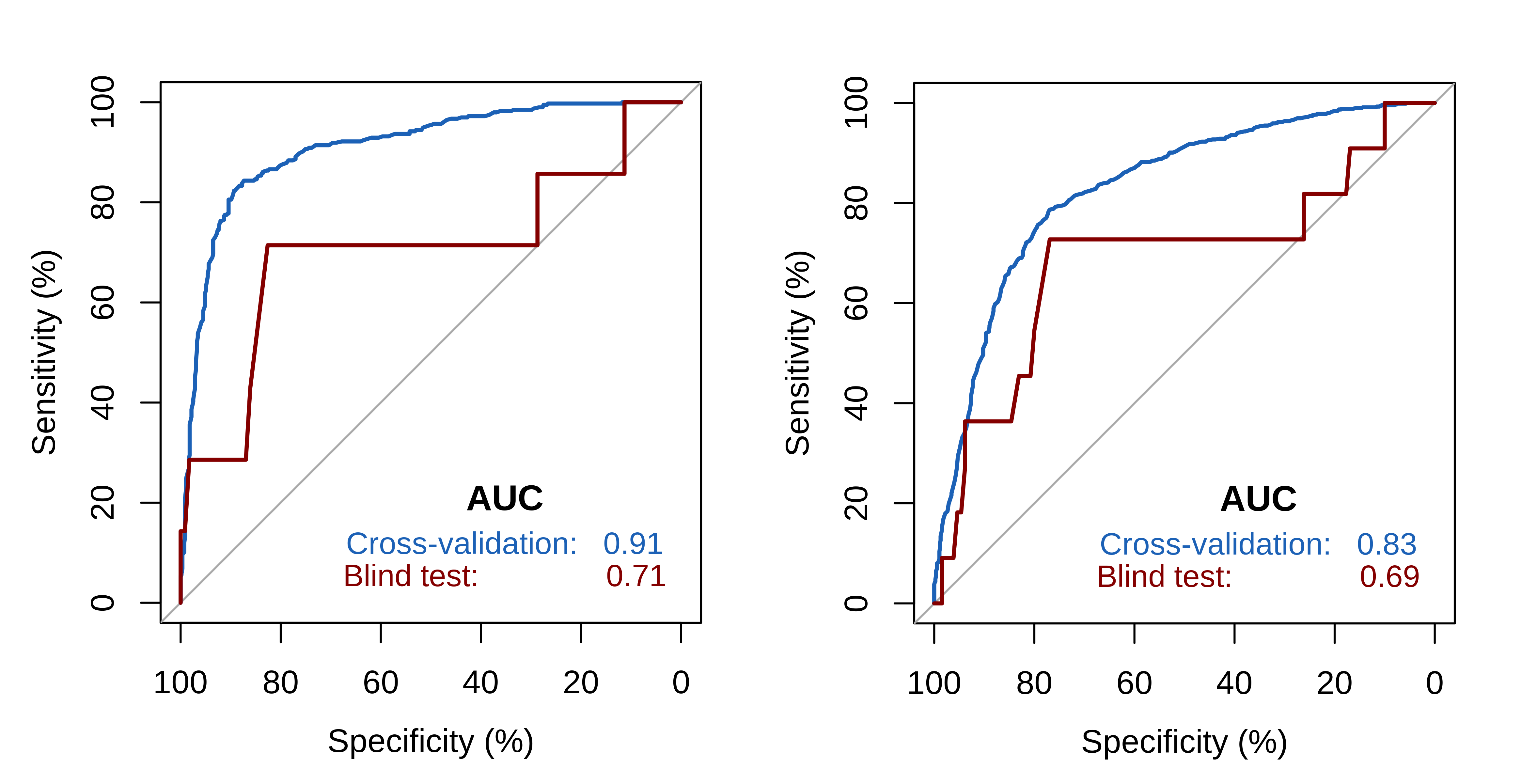


**Figure S4.** Receiver operating characteristic (ROC) plot of the classification prediction of single-(cross-validation) and multiple-point mutations (blind test) in mmCSM-NA. Our method achieved

an Area Under the ROC curve (AUC) of 0.91 and 0.90 under cross-validation and blind test,

respectively, when considering stabilising mutations those with ΔΔG > 0.5 kcal/mol and

destabilising mutations those with ΔΔG < -0.5 kcal/mol (left-hand plot). It achieved AUCs of 0.83

and 0.86 for cross-validation and blind test, respectively, when only considering the sign of the

change (right-hand plot).


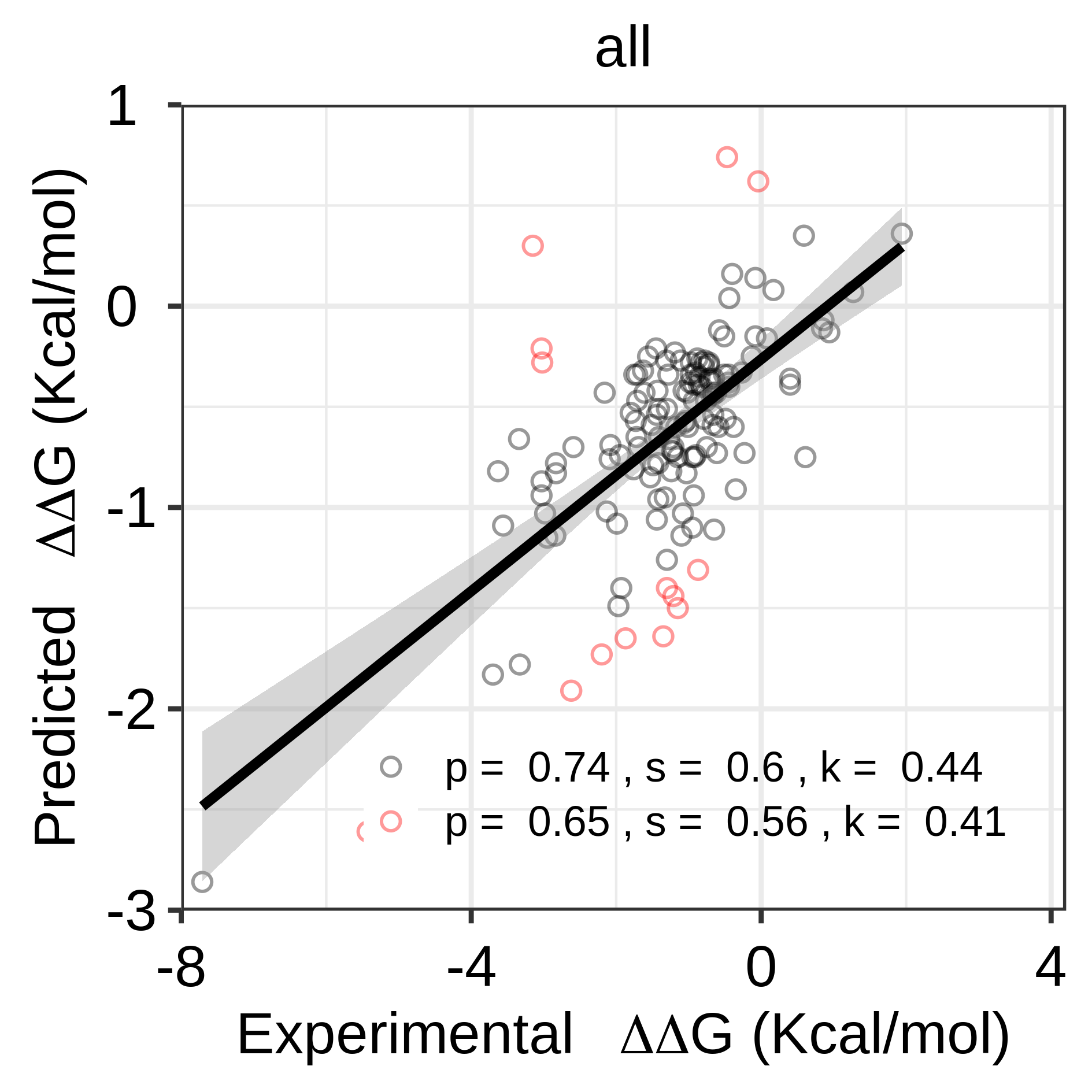
 **Figure S5.** Regression plot between the experimental and predicted changes in binding affinity (in Kcal/mol) during blind tests. mmCSM-NA obtained a Pearson’s correlation of 0.65 across the original data set.


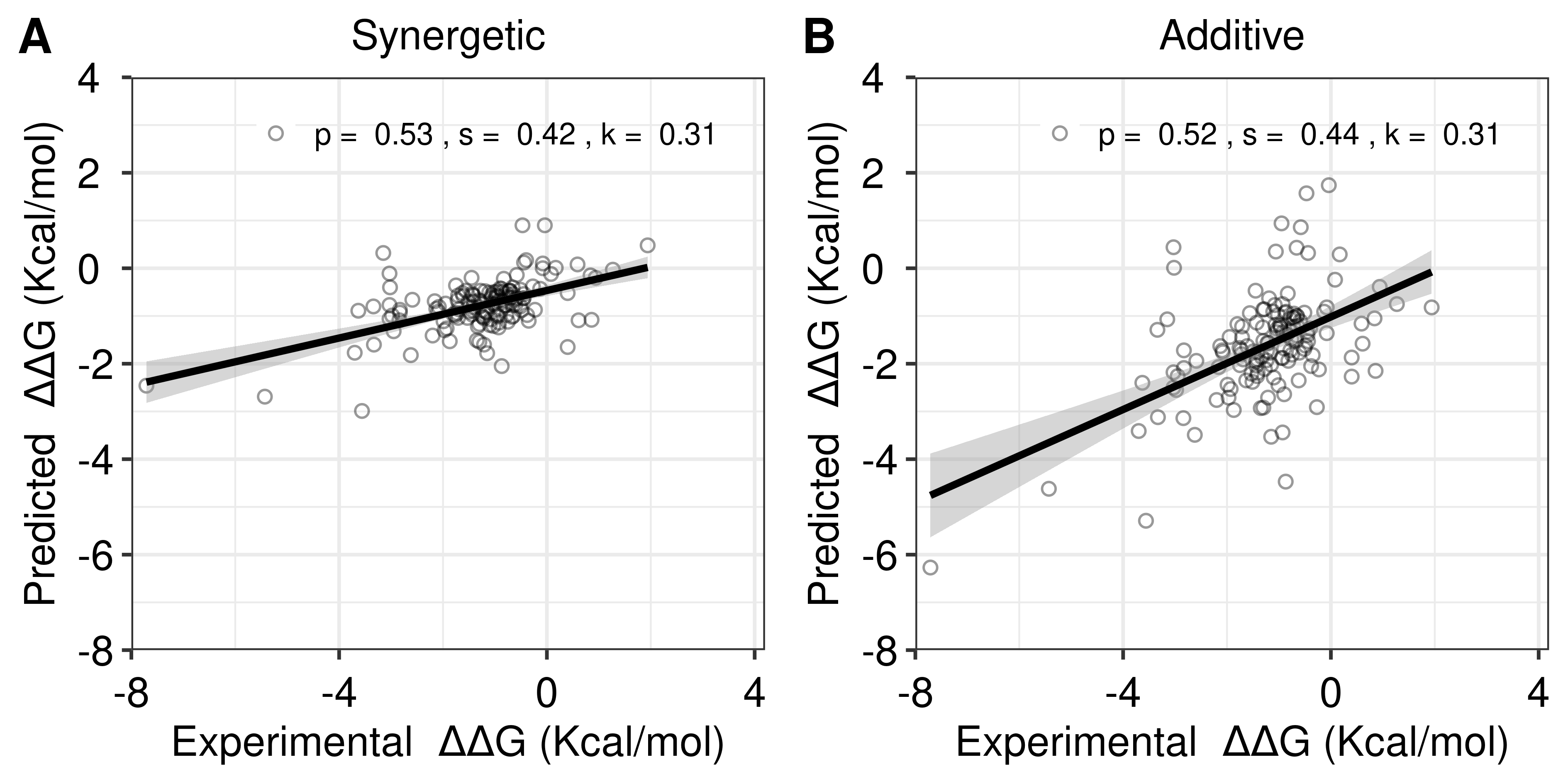
**Figure S6.** Regression plot between the experimental and predicted changes in binding affinity (in Kcal/mol) during blind tests with synergetic and additive effects.


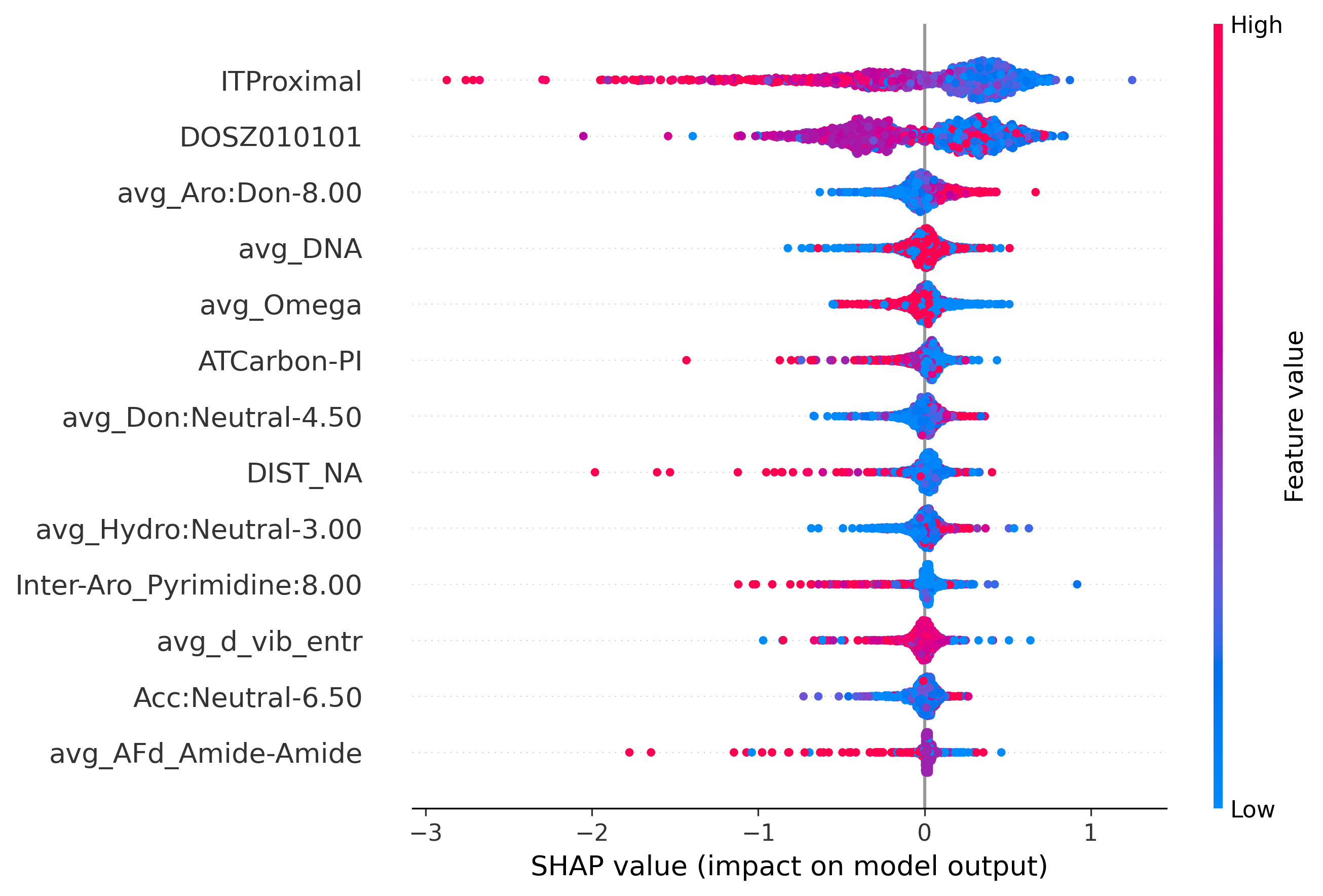


**Figure S7.** Feature importance of 13 selected features in the final model of mmCSM-NA. The high/low importances are represented in red and blue, respectively.


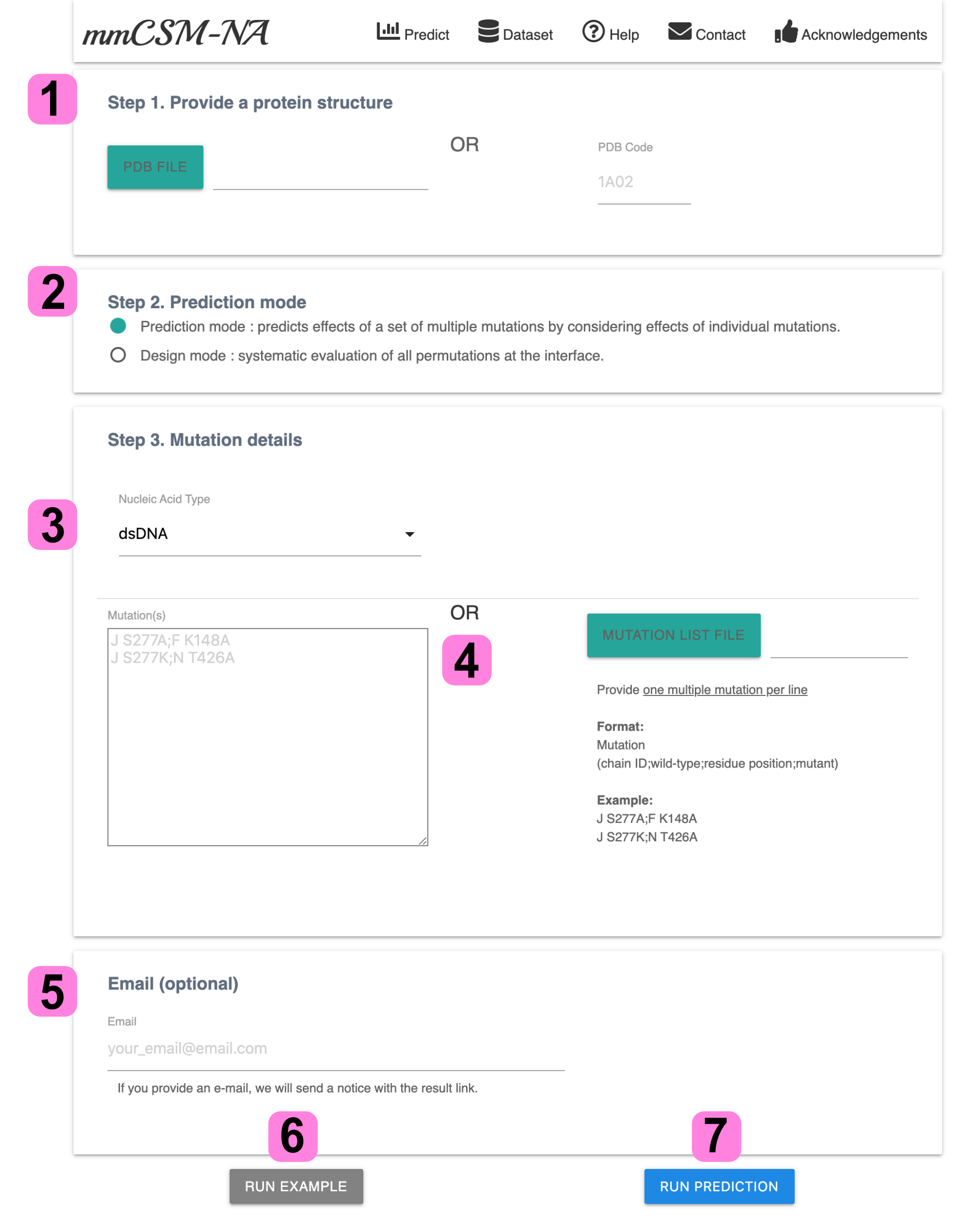
**Figure S8.** Job submission interface. Users should provide the protein–NA complex either by uploading a PDB file or providing a PDB accession code (1). User can choose between prediction mode or design mode (2). In the mutation details, users should specify nucleic acid type, namely single-stranded RNA (ssRNA), double-stranded RNA (dsRNA), single-stranded DNA (ssDNA), and double-stranded DNA (dsDNA) (3). Users can choose between submitting a single mutation, multiple mutations, or a list of mutations for analysis, as a separate file (4). It is optional to give the email address to get the a notification when the job is complete (5). Users can run an example (6) or run prediction to get the prediction on the given structure (7).


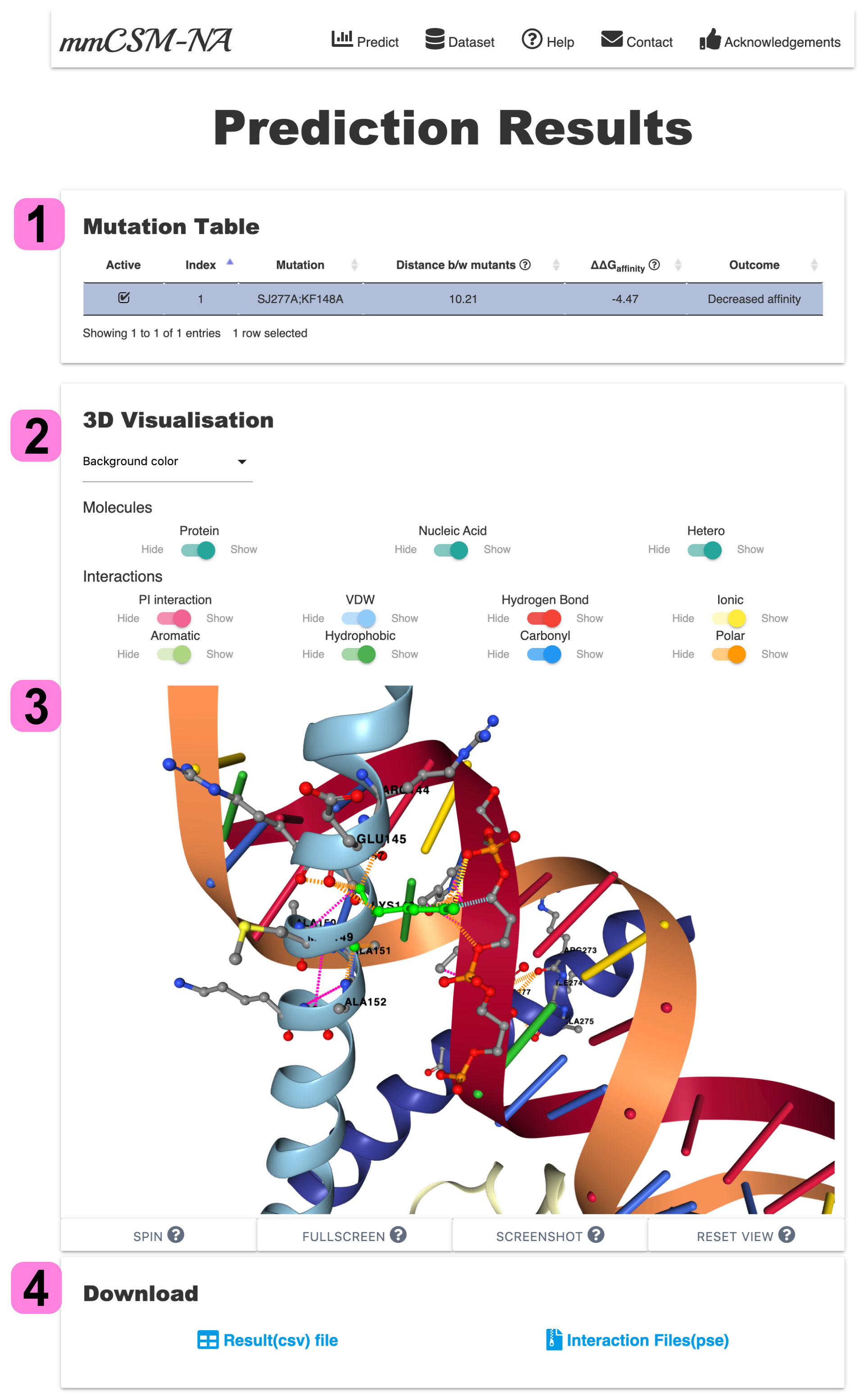
**Figure S9.** Web server results page for the predictions of single- and multiple-point mutations. The mutation table gives predicted change in affinity upon mutation (ΔΔG in kcal/mol) and distance among mutations. The interaction between protein and nucleic acid can be ticking in a checkbox (2), and visualized directly from the server (3). The mutation data and predictions are available for download in csv format as well as molecular interactions as Pymol session files (4).
